# Echocardiographic Evidence of Left Ventricular Dysfunction in COPD: Relationship with Disease Severity

**DOI:** 10.3390/medicina61071260

**Published:** 2025-07-11

**Authors:** Rounak Bhattacharjee, Tanushree Deb, Prosenjit Roy, Prithwiraj Bhattacharjee, Israel Maldonado Rosas, Shubhadeep Roychoudhury

**Affiliations:** 1 Department of Medicine, Lumding Civil Hospital, Lumding 782447, India; 2 Department of Medicine, Pragjyotishpur Medical College and Hospital, Guwahati 781009, India; 3 Department of Medicine, Nalbari Medical College and Hospital, Nalbari 781351, India; 4 Department of Medicine, Silchar Medical College and Hospital, Silchar 788014, India; 5 Citmer Reproductive Medicine, Mexico City 11520, Mexico; 6 Department of Life Science and Bioinformatics, Assam University, Silchar 788011, India

**Keywords:** chronic obstructive pulmonary disease, diastolic dysfunction, systolic dysfunction, GOLD stage, exacerbation, hypoxemia

## Abstract

*Background and Objectives*: Chronic obstructive pulmonary disease (COPD) significantly impacts morbidity and mortality, often due to cardiovascular comorbidities that are frequently overlooked. This study examines the prevalence of left ventricular dysfunction in COPD patients and its association with disease severity, hypoxemia, and exacerbation frequency. *Materials and Methods*: COPD patients (*n* = 114) were evaluated using spirometry and transthoracic echocardiography. Statistical analysis utilized Student’s *t*-test, chi-square test, and multivariable logistic regression with 1000 bootstrapping iterations, considering *p* < 0.05 as significant differences. *Results*: Most patients were classified as Global Initiative for Chronic Obstructive Lung Disease (GOLD) stage III (40.4%) and stage IV (44.7%). Diastolic dysfunction was present in 67.5% of the patients (Grade 1: 53.5%, Grade 2: 13. 2%, Grade 3: 0.0.9%), while 18.4% exhibited systolic dysfunction (LVEF < 50%). The prevalence of diastolic dysfunction increased significantly, from 41.2% in GOLD stage II to 92. 2% in GOLD stage IV (*p* < 0.001). Independent predictors of diastolic dysfunction included GOLD stage IV (Odds Ratio [OR]: 5.39, 95% Confidence Interval [CI]: 1. 42–23.35, *p* < 0.001), older age (OR: 1.02 per year, 95% CI: 1.01–1.04, *p* = 0.025), and a history of frequent exacerbations (OR: 1.09 per event, 95% CI: 1.01–1.17, *p* = 0.039). Systolic dysfunction correlated significantly with GOLD stage IV (OR: 1.83, *p* = 0.014), oxygen saturation below 88% (OR: 3.12, *p* = 0.036), and having three or more exacerbations (OR: 4.18, *p* = 0.008). *Conclusions*: This study reveals a high prevalence of left ventricular dysfunction in COPD patients, linked to disease severity, hypoxemia, and frequent exacerbations. It supports incorporating complementary echocardiographic assessments in managing advanced COPD, especially for those with frequent exacerbations or oxygen desaturation.

## 1. Introduction

Chronic obstructive pulmonary disease (COPD) represents a significant global health burden, currently ranking as the third leading cause of mortality worldwide [1]. Beyond the characteristic airflow limitation, COPD is increasingly recognized as a complex multisystem disorder with significant extrapulmonary manifestations, particularly cardiovascular comorbidities [2,3]. These comorbidities substantially worsen clinical outcomes and contribute to increased hospitalization rates and mortality.

The cardiovascular manifestations of COPD extend beyond shared risk factors such as aging and tobacco exposure. Current evidence suggests that systemic inflammation, oxidative stress, chronic hypoxemia, and autonomic dysregulation directly contribute to myocardial dysfunction in COPD [4,5]. A recent study showed that a substantial number of patients with COPD had cardiac abnormalities that had not been identified before. In addition to a simultaneous burden of coronary artery disease and atrial fibrillation, the investigators found undiscovered left ventricular systolic dysfunction, indicating an underestimated overlap between cardiovascular and respiratory pathology in that cohort [5].

The pathophysiological impact of COPD on cardiac function involves multiple mechanisms. Chronic hypoxemia and pulmonary vascular remodeling impair myocardial oxygenation and promote ventricular remodeling [6,7]. Alterations in autonomic regulation, as evidenced by impaired heart rate variability, may precede overt structural cardiac changes [7]. Left ventricular diastolic and systolic dysfunction represent clinically significant consequences, associated with reduced exercise capacity, increased exacerbation frequency, and higher mortality [8,9,10]. Despite these established associations, cardiac abnormalities frequently remain undetected in COPD due to overlapping symptomatology and the absence of routine cardiovascular assessment in current management protocols [11,12]. Transthoracic echocardiography provides a non-invasive, widely accessible tool for evaluating left ventricular function; however, it still remains underexplored in COPD populations [13,14,15]. Recently, a hybrid ensemble method for predicting cardiovascular risk—HeartEnsembleNet—reported an accuracy of 92.95% and a precision of 93.08%. When considering similar predictive models for diseases like COPD, the methodological framework of this hybrid ensemble method may be worth comparing with the existing models for COPD, considering its potential implications in clinical practice [16].

The present study aimed to investigate echocardiographic evidence of left ventricular dysfunction—both systolic and diastolic—in patients with COPD attending a tertiary care hospital in Silchar, India. The population, characterized by a high prevalence of advanced disease stages and significant biomass fuel exposure, offers a valuable opportunity to examine the relationship between COPD severity, hypoxemia, exacerbation frequency, and cardiac function. The goal of this investigation is to identify key clinical and pathophysiological factors associated with cardiac issues. It is expected that the findings will support the need to include regular cardiac examinations in the management of COPD, allowing for the early detection and treatment of cardiovascular comorbidities in this high-risk group.

## 2. Materials and Methods

### 2.1. Study Design and Setting

This prospective observational study was conducted at the Department of Medicine, Silchar Medical College and Hospital, Silchar, India, between 1 March 2023 and 29 February 2024. The Institutional Human Ethics Committee (IHEC) of Silchar Medical College and Hospital approved the study protocol. All participants provided written informed consent before enrollment. The study was conducted in accordance with the Declaration of Helsinki and local regulatory standards.

### 2.2. Patient Selection

Patients were consecutively recruited from the inpatient and outpatient services of the Department of Medicine. A diagnosis of COPD was established based on the Global Initiative for Chronic Obstructive Lung Disease (GOLD) 2023 criteria [2], which incorporated clinical symptoms, exposure history (including smoking or biomass fuel), and spirometric evidence of persistent airflow limitation, i.e., post-bronchodilator forced expiratory volume in 1 s/forced vital capacity ratio <0.70 (FEV_1_/FVC ratio <0.70).

Inclusion criteria comprised age ≥40 years, confirmed COPD diagnosis as per GOLD 2023 guidelines, adequate spirometry performance, and provision of written informed consent. Exclusion criteria included asthma or other primary respiratory disorders, known cardiac diseases (valvular heart disease, congenital defects, cardiomyopathies), recent acute coronary syndrome (within the past 3 months), severe systemic illness affecting cardiac function, pregnancy or lactation, inability to perform spirometry, and declined consent.

### 2.3. Clinical and Pulmonary Assessment

Each participant underwent a comprehensive clinical evaluation, which included demographic data, comorbidities, respiratory symptoms, smoking status (in terms of pack-years), and biomass fuel exposure (among female participants). Vital parameters were documented, including respiratory rate, blood pressure, and resting oxygen saturation (measured via pulse oximeter on room air). The participants’ exacerbation history over the preceding 12 months was recorded.

Pulmonary function testing was performed using a calibrated spirometer (Spirolab III, MIR, Italy) [17]. Post-bronchodilator spirometry (after 400 µg salbutamol via MDI with spacer) was used to assess FEV_1_, FVC, FEV_1_/FVC ratio, and FEV_1_% predicted.

Disease severity was classified according to GOLD stages:—GOLD I: FEV_1_ ≥ 80%, GOLD II: 50–79%, GOLD III: 30–49%, and GOLD IV: < 30%.

### 2.4. Laboratory Investigations

Peripheral blood samples were analyzed for complete blood count, erythrocyte sedimentation rate (ESR), alanine transaminase (ALT), aspartate aminotransferase (AST), random blood sugar, serum creatinine, and blood urea to assess systemic comorbidities and ensure eligibility.

### 2.5. Echocardiographic Evaluation

All patients underwent transthoracic echocardiography performed by a single experienced cardiologist blinded to clinical data, using a Philips EPIQ 7C system. Standard parasternal and apical views were obtained in accordance with the American Society of Echocardiography (ASE) guidelines [18].

Left ventricular systolic function was assessed using Simpson’s biplane method. Left ventricular systolic dysfunction (LVSD) was defined as left ventricular ejection fraction (LVEF) <50%. Diastolic function was evaluated according to ASE criteria [19], including: mitral inflow (E/A ratio and deceleration time), tissue doppler (septal and lateral e’ velocities), and average E/e’ ratio (with grading as Grade 1: E/A ≤ 0.8, E/e’ < 10; Grade 2: E/A 0.8–2.0, E/e’ 10–14; and Grade 3: E/A ≥ 2.0, E/e’ >14).

When indices were discordant, left atrium (LA) volume, tricuspid regurgitation velocity (TRV), and pulmonary vein flow were used for classification. Additional parameters included LA volume index, left ventricular mass index (LVMi), right ventricular (RV) function, and pulmonary artery systolic pressure.

### 2.6. Statistical Analysis

The data were analyzed using R software version 4.2.0 (R Foundation for Statistical Computing, Vienna, Austria). Continuous variables were expressed as mean ± standard deviation (SD) or median (IQR) and compared using Student’s *t*-test or Mann-Whitney U test as appropriate. Categorical variables were expressed as frequencies and percentages and analyzed using the Chi-square or Fisher’s exact test. Correlations were assessed using Pearson or Spearman coefficients.

Multivariable logistic regression was performed to identify independent predictors of LV dysfunction. Bootstrapping (1000 iterations) was employed for model stability [20]. Benjamini–Hochberg correction was applied to account for multiple comparisons [21], and unadjusted as well as corrected *p*-values were reported. Statistical significance was set at *p* < 0.05. No imputation was performed, as there were no missing data.

## 3. Results

### 3.1. Demographic and Clinical Characteristics

A total of 114 patients with COPD were enrolled, with a mean age of 62.1 ± 9.5 years. The majority were male (70.2%) and belonged to the 51–70 age group (70.2%). Most patients were smokers (88.6%), with a mean smoking exposure of 25.0 ± 8.3 pack-years. Among female participants, 26.5% reported significant exposure to biomass fuel. Inhaler use was reported by 50.0% of patients. The most common symptoms were cough (79.8%), dyspnea (71.9%), fever (41.2%), and sputum production (38.6%). The mean respiratory rate was 27.1 ± 2.7 breaths/min, the mean oxygen saturation (SpO_2_) was 84.6 ± 5.0%, and the mean body mass index (BMI) was 19.3 ± 1.4 kg/m^2^. Exacerbation history in the previous year showed 29.8% participants had none, 27.2% had one, 21.1% had two, and 21.9% had three or more (Table 1).

### 3.2. COPD Severity and Lung Function

No patients were identified as being classified in GOLD stage I. In the cohort under study, GOLD stage II comprised 14.9% (*n* = 17) participants, GOLD stage III accounted for 40.4% (*n* = 46), and GOLD stage IV represented 44.7% (*n* = 51). The mean FEV_1_/FVC ratio was 53.6 ± 7.8%, while the mean predicted FEV_1_ was 34.6 ± 10.4%, indicating a patient population with moderate to severe chronic obstructive pulmonary disease (Table 2).

### 3.3. Prevalence of Left Ventricular Dysfunction

Diastolic dysfunction was identified in 67.5% (*n* = 77) patients, with Grade 1 being the most prevalent (53.5%, *n* = 61), followed by Grade 2 (13.2%, *n* = 15) and Grade 3 (0.9%, *n* = 1). The remaining 32.5% (*n* = 37) exhibited normal diastolic function. Systolic dysfunction (LVEF < 50%) was detected in 18.4% (*n* = 21), with a mean LVEF of 44.8 ± 3.2% for those affected, compared to 58.3 ± 4.7% in unaffected individuals.

### 3.4. Association Between COPD Severity and Cardiac Dysfunction

**Figure 1:**
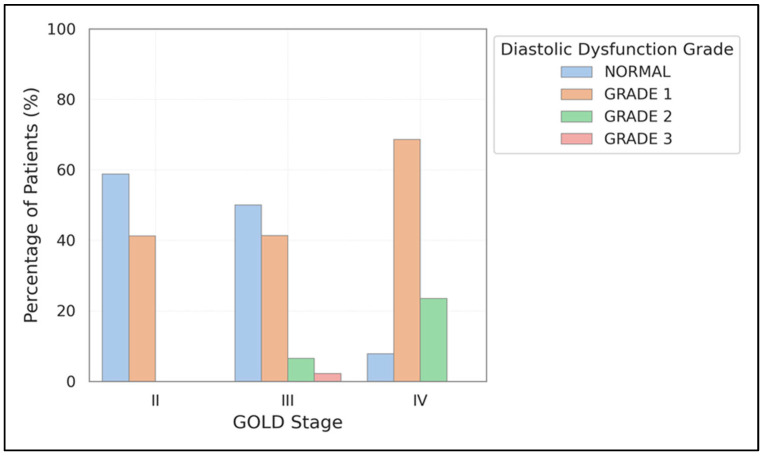
Prevalence of diastolic dysfunction grades across GOLD stages. The graph shows increasing prevalence of diastolic dysfunction with worsening COPD severity, with Grade 1 and Grade 2 dysfunction becoming more common in advanced disease stages.

The current study revealed a notable link between the advancement of the GOLD stage and the prevalence of diastolic and systolic dysfunction. The prevalence of diastolic dysfunction increased from 41.2% in GOLD II to 92.2% in GOLD IV (χ^2^ = 24.32, *p* < 0.001). Similarly, systolic dysfunction rose from 5.9% to 27.5% within the same stages (χ^2^ = 9.62, *p* = 0.008) (Figure 1).

### 3.5. Subgroup Analyses

#### 3.5.1. Smoking Status

**Figure 2:**
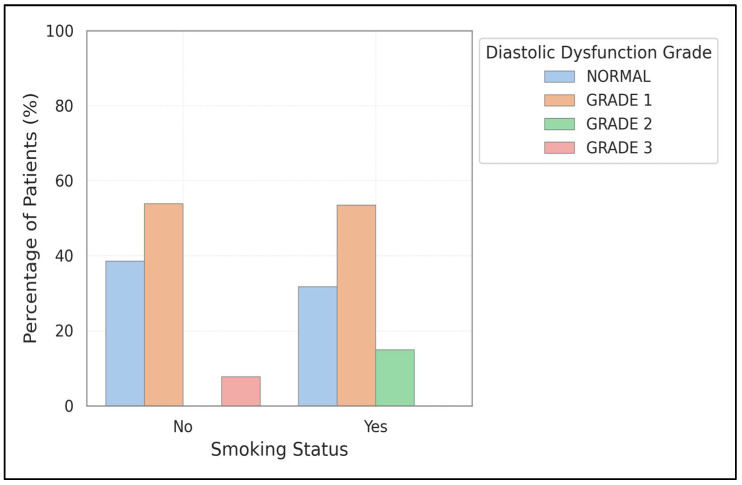
Comparison of diastolic dysfunction prevalence and severity between smokers and non-smokers. The chart shows higher overall prevalence and greater severity of diastolic dysfunction in smokers compared to non-smokers.

Diastolic dysfunction was more frequently observed in smokers (72.5%) compared to non-smokers (55.9%), although this difference was not statistically significant (*p* = 0.077). Notably, Grade 2 dysfunction was almost twice as prevalent in smokers (15.0% versus 8.8%). Systolic dysfunction was found in 20.0% of smokers and 14.7% of non-smokers. Figure 2 reinforces these findings and emphasizes the trend toward increased severity of diastolic dysfunction among smokers. Further details of diastolic and systolic dysfunction by smoking status can be found in Supplementary Figure S1.

#### 3.5.2. Oxygen Saturation

**Figure 3:**
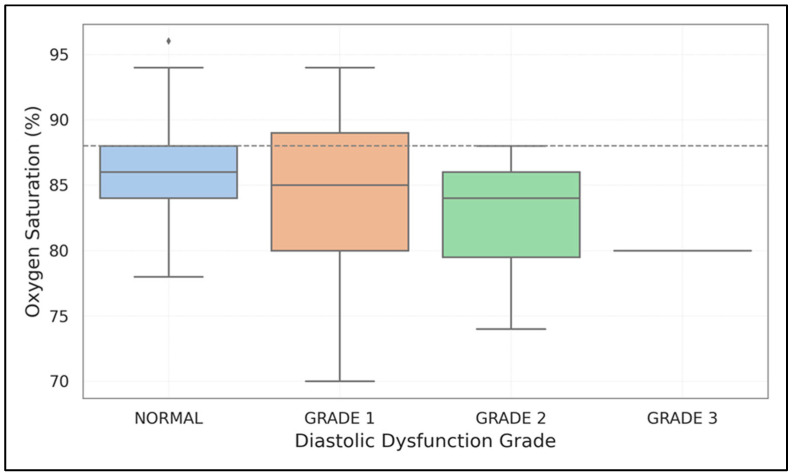
Relationship between oxygen saturation and diastolic dysfunction grades. The box plot demonstrates progressively lower SpO_2_ values with increasing severity of diastolic dysfunction, with a threshold of SpO_2_ < 88% highlighted.

Both forms of left ventricular dysfunction were linked to lower SpO_2_ levels. For diastolic dysfunction, the rates were 82.5 ± 6.1% compared to 86.9 ± 5.6% (*p* < 0.001); for systolic dysfunction, they were 80.2 ± 6.4% versus 84.7 ± 5.9% (*p* = 0.003). Figure 3 illustrates a decreasing trend in oxygen saturation as diastolic dysfunction progresses from Grade 1 to Grade 3, with a critical threshold at SpO_2_ < 88% highlighted.

#### 3.5.3. Exacerbation Frequency

**Figure 4:**
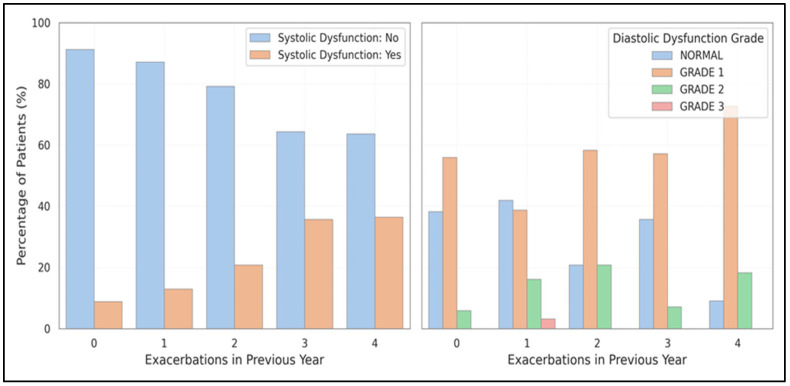
Illustrates the relationship between exacerbation frequency and left ventricular dysfunction, showing the dose-response relationship between the number of exacerbations in the past year and the prevalence of systolic and diastolic dysfunction.

The occurrence of systolic dysfunction increased from 8.8% (no exacerbations) to 36.0% (≥3 exacerbations) (χ^2^ = 7.95, *p* = 0.047). In contrast, diastolic dysfunction rose from 55.9% to 79.2%, although the statistical significance diminished after adjustment. Figure 4 depicts this dose-response pattern, highlighting how frequent exacerbations affect myocardial function.

#### 3.5.4. Pulmonary Function Correlation

Negative correlations were found between FEV_1_/FVC and the E/A ratio (r = −0.32, *p* < 0.001), as well as between FEV_1_% predicted and the E/e′ ratio (r = −0.31, *p* = 0.001). Additionally, LVEF showed a positive correlation with both FEV_1_/FVC and FEV_1_% predicted (r = 0.27–0.30, *p* ≤ 0.004), indicating that worsening airflow obstruction is linked to more severe left ventricular impairment.

### 3.6. Multivariate Analysis

**Figure 5:**
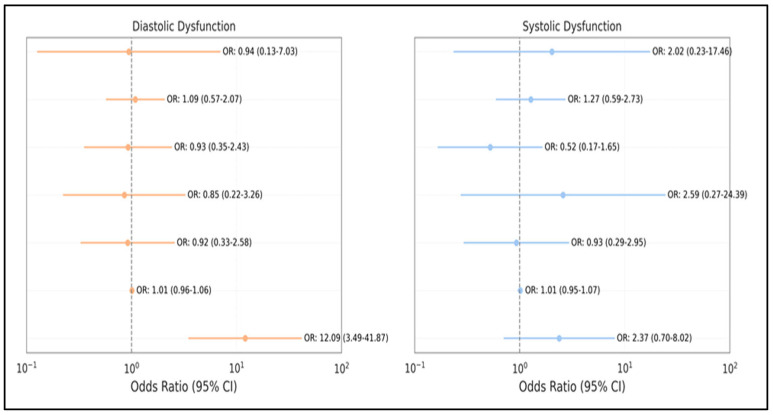
Forest plot of independent predictors for left ventricular dysfunction. The plot displays odds ratios with 95% confidence intervals for significant predictors of both diastolic and systolic dysfunction identified in multivariate analysis.

Among the candidate predictors tested, only GOLD stage IV was independently associated with diastolic dysfunction (OR: 12.09, 95% CI: 3.49–41.87, *p* < 0.001). None of the predictors reached statistical significance for systolic dysfunction in the multivariate model, although GOLD stage IV (OR: 2.37, *p* = 0.164) and ≥3 exacerbations (OR: 2.02, *p* = 0.522) exhibited a consistent directional trend (Figure 5).

## 4. Discussion

The current study revealed a significant prevalence of left ventricular dysfunction in COPD patients, with 67.5% exhibiting diastolic dysfunction and 18.4% demonstrating systolic dysfunction. Both types of dysfunctions showed strong, statistically significant links to increasing severity of COPD (GOLD staging), diminished pulmonary function, oxygen desaturation, and frequent exacerbations. GOLD stage IV, older age, and frequency of exacerbations independently predicted diastolic dysfunction. In contrast, systolic dysfunction was independently linked to GOLD stage IV, oxygen saturation below 88%, and three or more exacerbations in the past year.

The prevalence of systolic dysfunction observed in our study aligns with that of Kibbler et al. [5], who recently noted a pooled rate of 15.8% in their meta-analysis. The slightly higher rate in our cohort (18.4%) probably reflects the higher concentration of patients in GOLD III and IV stages. Freixa et al. [22] and Huang et al. [23] also reported an increase in LV dysfunction in later stages of COPD, especially among GOLD IV patients. Our findings enhance this understanding through a representative cohort significantly leaning towards advanced disease, with a 92.2% prevalence of diastolic dysfunction in GOLD IV patients. The group with normal function significantly decreases in GOLD IV, highlighting the impact of advanced COPD on left ventricular relaxation.

Previous studies by Sabit et al. [24] and Funk et al. [25] identified significant correlations between FEV_1_ and echocardiographic indicators of LV diastolic dysfunction. Our results corroborate with significant relationships between both FEV_1_% predicted and the E/e′ ratio. Furthermore, our data support the influence of oxygen desaturation on cardiac function, consistent with earlier research connecting chronic hypoxemia to myocardial dysfunction and pulmonary hypertension [26,27].

In earlier studies, MacDonald et al. [27] and Patel et al. [28] emphasized the connection between exacerbations and cardiac outcomes. The current study’s findings reinforce this connection, showing a clear dose-response relationship between exacerbation frequency and diastolic and systolic dysfunction. These results highlight the cardiovascular burden associated with exacerbation-prone COPD phenotypes.

Multiple overlapping mechanisms might clarify the observed relationships. Cigarette smoking leads to oxidative stress, systemic inflammation, and direct myocardial damage [28]. Despite no significant differences in overall cardiac dysfunction rates between smokers and non-smokers, smokers in our study displayed more severe (Grade 2) diastolic dysfunction.

Hypoxemia hinders myocardial oxygen delivery and exacerbates systolic and diastolic dysfunction by directly impacting myocardial relaxation, increased afterload, and remodeling of pulmonary vasculature [26,27]. In our group, lower SpO_2_ was linked to both types of dysfunctions, with patients having saturations above 92% showing significantly reduced rates of cardiac impairment.

Systemic inflammation—long seen as a hallmark of COPD—can drive myocardial fibrosis and endothelial dysfunction, mechanisms that may contribute to diastolic stiffness and subclinical systolic failure [29,30]. In advanced COPD, pulmonary hyperinflation further limits cardiac filling by decreasing venous return and modifying ventricular interdependence [31,32].

Moreover, autonomic dysfunction in COPD, characterized by altered heart rate variability and increased sympathetic tone, may facilitate myocardial remodeling and be a factor in LV dysfunction [33]. Another previous study suggests that frequent exacerbations function as acute “cardiac stress tests”, emphasizing the need for more research into the cardiovascular impacts of these events [34].

Given the common occurrence of LV dysfunction and its link to disease severity, exacerbation, and hypoxemia, it may be an important complementary evaluation of cardiac health in COPD patients, especially those classified as GOLD stages III and IV. Transthoracic echocardiography is a cost-effective and readily available method for detecting subclinical cardiac issues. Identifying diastolic and systolic dysfunction early may inform pulmonary and cardiovascular treatment strategies.

Integrating complementary cardiopulmonary assessments into COPD management can impact treatment choices. Optimizing bronchodilator therapy can alleviate hyperinflation and enhance venous return, which may positively affect cardiac function. Clinicians should also be mindful of the cardiovascular safety profiles of medications prescribed for COPD.

Additionally, preventing exacerbations—through the guideline-based application of inhaled therapies, pulmonary rehabilitation, and vaccination—could help limit cardiovascular complications [35]. Long-term oxygen therapy has been demonstrated to enhance survival rates and cardiac function for patients with hypoxemia [36]. Integrative care models that include pulmonologists and cardiologists may be especially beneficial for this high-risk group.

This study possesses several significant strengths, including a strong representation of GOLD III–IV patients and standardized evaluations of pulmonary and cardiac function. Blinding the echocardiography operator to the clinical status helps minimize the risk of detection bias. Our research showed that 67.5% of COPD patients exhibit diastolic dysfunction, with 18.4% also experiencing systolic dysfunction. This underscores the importance of early detection and proactive management of cardiovascular issues in this group. Using machine learning can improve early diagnosis by revealing complex, non-linear connections among variables such as spirometric indices, echocardiographic data, biomarkers, and comorbidities—patterns that traditional statistical methods might overlook [16,37].

While the study offers valuable insights, it has some limitations. For instance, it did not fully account for conditions such as diabetes, high blood pressure, and kidney issues, which may also affect heart performance outside of COPD. The analysis of pulmonary hypertension and hypoxemia was constrained because PASP measurements were not always available. Furthermore, without a healthy control group matched for age and sex, it is challenging to determine precisely how COPD affects the heart. As a cross-sectional observational study, it cannot establish causality either. The findings may also vary since they are based on a real-world clinical population. Future studies should include well-matched control groups to clarify the specific impact of COPD on the heart. Long-term research is necessary to explore causal relationships and track the progression of heart dysfunction. Gathering detailed data on other health conditions and ensuring accurate PASP measurement, particularly in relation to hypoxemia and pulmonary hypertension, will enhance our understanding of the heart–lung connection. Ultimately, early detection of heart involvement in COPD could improve risk assessment and enable more timely treatment.

## 5. Conclusions

This study provides strong evidence of a significant burden of left ventricular (LV) dysfunction in COPD patients, especially in advanced stages. Diastolic dysfunction was observed in roughly two-thirds of participants, while nearly 20% showed systolic dysfunction; both were linked to worse pulmonary function, hypoxemia, and frequent exacerbations. These findings underscore the importance of proactive cardiovascular screening in COPD patients, particularly those with severe disease, oxygen desaturation, or exacerbation-prone features. Early detection of cardiac issues—even without traditional symptoms—may enable more personalized and integrated treatment strategies. The connections found support a multifactorial pathophysiology involving systemic inflammation, hypoxemia, autonomic imbalance, and direct pulmonary–cardiac interactions. Future research should focus on prospective, longitudinal studies to identify causative factors and assess as to whether early cardiac screening and targeted therapies can improve outcomes for such a high-risk group.

## Figures and Tables

**Table 1:** Demographic and clinical characteristics of study participants (*n* = 114).

Characteristic	Value
Age (years)	62.1 ± 9.5 (mean ± SD)
Male (*n* = 80)	70.2%
Female (*n* = 34)	29.8%
Body mass index (BMI) (kg/m^2^)	21.4 ± 3.8 (mean ± SD)
Current/former smoker (*n* = 80)	70.2%
Never smoker (*n* = 34)	29.8%
Pack-years (smokers)	25.0 ± 8.3 (mean ± SD)
Biomass exposure (females) (*n* = 9)	26.5%
Cough (*n* = 91)	79.8%
Dyspnea (*n* = 82)	71.9%
Fever (*n* = 47)	41.2%
Sputum production (*n* = 44)	38.6%
Respiratory rate (breaths/min)	27.1 ± 3.4 (mean ± SD)
Mean oxygen saturation (SpO_2_) (%)	83.9 ± 6.2 (mean ± SD)
Number of exacerbations in the previous year	
None (*n* = 34)	29.8%
One (*n* = 31)	27.2%
Two (*n* = 24)	21.1%
Three or more (*n* = 25)	21.9%
Inhaler use (*n* = 57)	50.0%

SD: standard deviation.

**Table 2:** Pulmonary Function and GOLD Classification (*n* = 114).

Parameter	Value
Forced expiratory volume in 1 s (FEV_1_) (L)	1.02 ± 0.38 (mean ± SD)
FEV_1_% predicted	34.6 ± 10.4 (mean ± SD)
Forced vital capacity (FVC) (L)	1.91 ± 0.59 (mean ± SD)
FEV_1_/FVC ratio (%)	53.6 ± 7.8 (mean ± SD)
Classification of participants based on the GOLD guideline	
GOLD I (*n* = 0)	0.0%
GOLD II (*n* = 17)	14.9%
GOLD III (*n* = 46)	40.4%
GOLD IV (*n* = 51)	44.7%

SD: standard deviation.

## Data Availability

The original contributions presented in this study are included in the article/Supplementary Material. Further inquiries can be directed to the corresponding authors.

## Supplementary Materials

The following supporting information can be downloaded at: https://www.mdpi.com/article/10.3390/medicina61071260/s1, Figure S1: Cardiac Dysfunction by Smoking Status.

## Abbreviations

IHEC	The Institutional Human Ethics Committee
GOLD	Global Initiative for Chronic Obstructive Lung Disease
COPD	Chronic obstructive pulmonary disease
FEV_1_	Forced expiratory volume in 1 s
FVC	Forced vital capacity
ESR	Erythrocyte sedimentation rate
ALT	Alanine transaminase
AST	Aspartate aminotransferase
ASE	American Society of Echocardiography
LVSD	Left ventricular systolic dysfunction
LVEF	Left ventricular ejection fraction
SpO_2_	Peripheral oxygen saturation
BMI	Body mass index
LA	Left atrium
TRV	Tricuspid regurgitation velocity
LVMi	Left ventricular mass index
RV	Right ventricular
LV	Left ventricular
