# Supplementary material for: Echocardiographic Evidence of Left Ventricular Dysfunction in COPD: Relationship with Disease Severity
Source: Medicina (Kaunas). 2025 Jul 11;61(7):1260. doi: 10.3390/medicina61071260 (PMC12301051; doi:10.3390/medicina61071260)
Supplement: Supplementary file 1 [file medicina-61-01260-s001.zip › medicina-3688504-supplementary.pdf]

Supplementary Materials

Subgroup Analyses:

Oxygen Saturation

Both types of LV dysfunction were associated with lower SpO_2. Diastolic dysfunction: 82.5 ± 6.1% vs. 86.9 ± 5.6% ($p < 0.001$); Systolic dysfunction: 80.2 ± 6.4% vs. 84.7 ± 5.9% ($p = 0.003$).

Figure 2 illustrates a downward trend in oxygen saturation as diastolic dysfunction progresses from Grade 1 to Grade 3, with a visual threshold of SpO_2 <88% emphasized.

Exacerbation Frequency

The prevalence of systolic dysfunction increased from 8.8% (no exacerbations) to 36.0% (≥3 exacerbations) ($\chi^2 = 7.95$, $p = 0.047$). Diastolic dysfunction also increased (from 55.9% to 79.2%), though statistical significance was not retained after correction.

Smoking Status

Diastolic dysfunction was more prevalent among smokers (68.3%) compared to non-smokers (61.5%), though this difference did not reach statistical significance ($p = 0.077$). Notably, Grade 2 dysfunction was observed in 14.9% of smokers but was absent among non-smokers. Systolic dysfunction was present in 19.8% of smokers versus 7.7% of non-smokers, reinforcing a possible link between smoking exposure and myocardial impairment.

These patterns are visualised in Supplementary **Figure S1**, which illustrates the distribution of cardiac dysfunction grades by smoking status.

Pulmonary Function Correlation

Negative correlations were seen between FEV_1/FVC and E/A ratio ($r = -0.32$, $p < 0.001$), and FEV_1% predicted and E/e′ ratio ($r = -0.31$, $p = 0.001$). LVEF correlated positively with both FEV_1/FVC and FEV_1% predicted ($r = 0.27$–0.30, $p \leq 0.004$), suggesting worsening airflow obstruction is associated with more pronounced LV dysfunction.

Figure S1. ***Cardiac Dysfunction by Smoking Status***

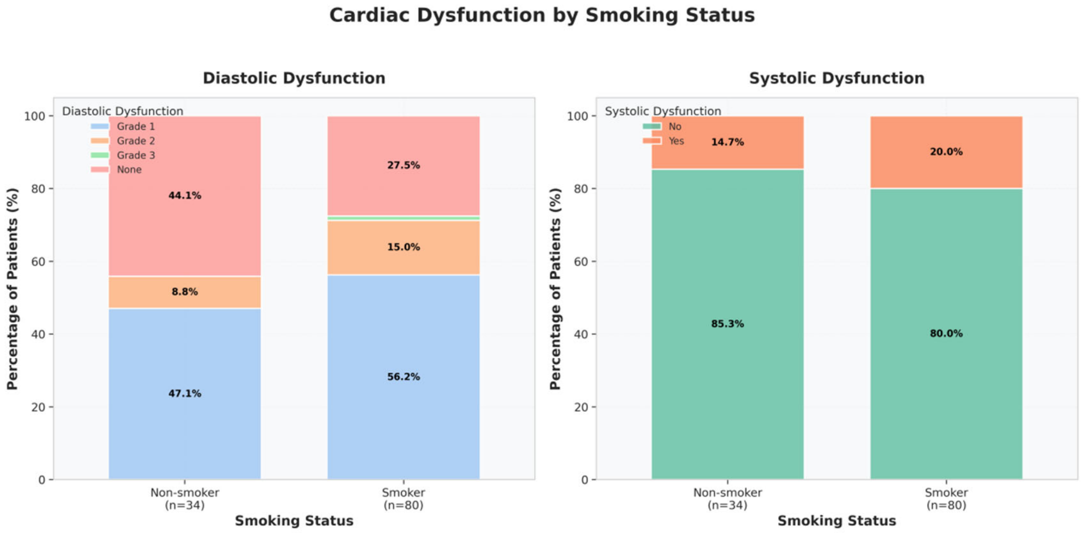

Side-by-side bar charts comparing the prevalence of diastolic and systolic dysfunction among smokers (n = 80) and non-smokers (n = 34). Smokers exhibited a higher proportion of Grade 2 diastolic dysfunction (14.9%) and a greater overall prevalence of systolic dysfunction (19.8% vs. 7.7%). These trends support a potential exacerbating effect of smoking on cardiac impairment in COPD.
